# A LONGITUDINAL INVESTIGATION OF ALCOHOL USAGE AND COGNITIVE OUTCOMES AMONG HISPANIC/LATINO ADULTS

**DOI:** 10.1093/geroni/igae098.2248

**Published:** 2024-12-31

**Authors:** Armando Lemus, Carlos Araujo-Menendez, Rubi Carpio, Ariana Stickel

**Affiliations:** San Diego State University, San Diego, California, United States; SDSU/UC San Diego, San Diego, California, United States; San Diego State University, San Diego, California, United States; San Diego State University, San Diego State University, California, United States

## Abstract

High alcohol use may lead to cognitive deficits and is recognized as a modifiable risk factor for dementia. However, recent cross-sectional research is contradictory: low to moderate alcohol may be protective against cognitive performance. The present study aimed to investigate the associations between alcohol use and cognitive decline within Hispanic/Latino adults, a community often underrepresented in the research field. Participants included 336 self-identified Hispanic/Latino adults (67% female; ages 65 - 92 years, M = 73.29 years, SD=5.5) without dementia from the National Alzheimer’s Coordinating Center dataset collected from 33 sites between 2015 – 2022. Alcohol use was assessed through self-reported consumption of any alcohol in the past 3 months (yes or no). Outcomes included cognitive tests administered to participants at a minimum of two visits. Using linear mixed models, we analyzed the relationships between alcohol use and cognitive trajectories, including possible modifications by sex and depressive symptoms (as assessed by the Geriatric Depression Scale), while adjusting for age, education, and cognitive status at baseline. Our results indicate that individuals who consumed alcohol performed better at baseline, (all F’s ≥ 6.187, all p’s < 0.01) but declined at the same rate across all cognitive tests, except for language. We did not detect significant modifications. Our results suggest that alcohol use is not protective against cognitive aging among Latino adults. However, additional measures (e.g., number of binges in a month) are needed to better understand its impact on dementia risk.

